# Adverse Clinical Outcomes Among Patients With Acute Low-risk Pulmonary Embolism and Concerning Computed Tomography Imaging Findings

**DOI:** 10.1001/jamanetworkopen.2023.11455

**Published:** 2023-05-31

**Authors:** Connor O’Hare, Kelsey A. Grace, William J. Schaeffer, S. Nabeel Hyder, Michael Stover, Amber L. Liles, Minhaj S. Khaja, James A. Cranford, Keith E. Kocher, Geoffrey D. Barnes, Colin F. Greineder

**Affiliations:** 1 Department of Emergency Medicine, University of Michigan, Ann Arbor; 2 Now with Department of Emergency Medicine, Medical College of Wisconsin, Milwaukee; 3 Department of Internal Medicine, Division of Cardiovascular Medicine, Frankel Cardiovascular Center, University of Michigan, Ann Arbor; 4 Department of Radiology, Division of Interventional Radiology, Frankel Cardiovascular Center, University of Michigan, Ann Arbor; 5 Department of Pharmacology, University of Michigan, Ann Arbor; 6 BioInterfaces Institute, University of Michigan, Ann Arbor

## Abstract

**Importance:**

Most patients presenting to US emergency departments (EDs) with acute pulmonary embolism (PE) are hospitalized, despite evidence from multiple society-based guidelines recommending consideration of outpatient treatment for those with low risk stratification scores. One barrier to outpatient treatment may be clinician concern regarding findings on PE-protocol computed tomography (CTPE), which are perceived as high risk but not incorporated into commonly used risk stratification tools.

**Objective:**

To evaluate the association of concerning CTPE findings with outcomes and treatment of patients in the ED with acute, low-risk PE.

**Design, Setting, and Participants:**

This cohort study used a registry of all acute PEs diagnosed in the adult ED of an academic medical center from October 10, 2016, to December 31, 2019. Acute PE cases were divided into high- and low-risk groups based on PE Severity Index (PESI) class alone or using a combination of PESI class and biomarker results. The low-risk group was further divided based on the presence of concerning CTPE findings: (1) bilateral central embolus, (2) right ventricle–to–left ventricle ratio greater than 1.0, (3) right ventricle enlargement, (4) septal abnormality, or (5) pulmonary infarction. Data analysis was conducted from June to October 2022.

**Main Outcomes and measures:**

The primary outcome was all-cause mortality at 7 and 30 days. Secondary outcomes included hospitalization, length of stay, need for intensive care, use of echocardiography and/or bedside ultrasonography, and activation of the PE response team (PERT) .

**Results:**

Of 817 patients (median [IQR] age, 58 [47-71] years; 417 (51.0%) female patients; 129 [15.8%] Black and 645 [78.9%] White patients) with acute PEs, 331 (40.5%) were low risk and 486 (59.5%) were high risk by PESI score. Clinical outcomes were similar for all low-risk patients, with no 30-day deaths in the low-risk group with concerning CTPE findings (0 of 151 patients) vs 4 of 180 (2.2%) in the low-risk group without concerning CTPE findings and 88 (18.1%) in the high-risk group (*P* < .001). Low-risk patients with concerning CTPE findings were less frequently discharged from the ED than those without concerning CTPE findings (3 [2.0%] vs 14 [7.8%]; *P* = .01) and had more frequent echocardiography (87 [57.6%] vs 49 [27.2%]; *P* < .001) and PERT activation for consideration of advanced therapies (34 [22.5%] vs 11 [6.1%]; *P* < .001).

**Conclusions and Relevance:**

In this single-center study, CTPE findings widely believed to confer high risk were associated with increased hospitalization and resource utilization in patients with low-risk PE but not short-term adverse clinical outcomes.

## Key Points

**Question:**

Are concerning computed tomography findings among patients seen in the emergency department with acute, low-risk pulmonary embolism (PE) (eg, saddle PE, right ventricular strain, pulmonary infarct) associated with differences in treatment and/or clinical outcomes?

**Findings:**

In this cohort study of 817 patients, concerning computed tomography findings were associated with increased hospitalization and resource utilization but not short-term adverse clinical outcomes.

**Meaning:**

These findings suggest that concerning computed tomography imaging findings may be a significant barrier to outpatient treatment among patients with otherwise low-risk acute PE.

## Teaser

This cohort study evaluates the association of concerning computed tomography (CT) findings with outcomes and treatment of patients in the emergency department with acute, low-risk pulmonary embolism (PE).

## Introduction

Of the approximately 250 000 patients diagnosed with acute pulmonary embolism (PE) in US emergency departments (EDs) each year, most are hospitalized, despite evidence from multiple studies and society-backed guidelines recommending consideration of discharge for the 25% to 50% with low risk stratification scores.^1,2,3,4,5,6,7,8,9^ One of the potential barriers to outpatient management may be clinician concern about findings on PE-protocol computed tomography (CTPE) that are perceived as high risk (eg, saddle PE, right ventricular [RV] strain, or pulmonary infarct) but not incorporated into commonly used risk stratification tools. Indeed, of the major strategies for identification of low-risk patients—the PE Severity Index (PESI) score,^10^ the simplified PESI (sPESI) score,^11^ the Hestia criteria,^12^ and the European Society of Cardiology (ESC) guidelines^7^—only the ESC guidelines include imaging findings, and these are limited to signs of RV enlargement or RV dysfunction (RVD).

In reality, little evidence is available regarding the prognostic value of CTPE imaging in patients with acute PE and low risk stratification scores. Early studies focused on echocardiographic markers, rather than CTPE findings, and were performed prior to the validation and widespread use of risk stratification tools.^13,14^ Later studies, which directly examined the prognostic value of CTPE findings, focused on their potential to predict decompensation in hemodynamically stable patients, rather than those with low risk stratification scores.^15,16^ Most recently, 2 meta-analyses^17,18^ produced conflicting results, with one finding higher rates of early adverse outcome in patients with low-risk PE and RV dysfunction on echocardiography or CTPE and the other finding no association between abnormal findings on CTPE and in-hospital or 30-day mortality.

These results aside, there remains concern among many emergency medicine (EM) clinicians that certain CTPE findings confer higher risk of adverse outcome, irrespective of risk stratification score. These include bilateral and centrally located emboli, extensive clot burden, pulmonary infarction, and radiographic evidence of RV strain (eg, septal abnormalities, RV dilation). In this study, we sought to understand the association of these so-called concerning CTPE findings with hospitalization, resource utilization, and patient outcomes, taking advantage of a large and well-curated registry of patients with acute PE diagnosed in the ED.

## Methods

### Construction of the University of Michigan Acute ED-PE Registry

This study was granted exemption from review and the requirement for informed consent by the institutional review board of the University of Michigan (UM). Construction of the UM registry of acute PEs diagnosed in the ED (ie, acute ED-PEs) is described in detail in eMethods in Supplement 1. Briefly, we started with positive PE cases identified by the Michigan Emergency Department Improvement Collaborative (MEDIC) and pooled these with cases identified by query of our electronic medical record (EMR). Each medical record was manually reviewed by 2 of us (S.N.H., C.O., K.A.G., and W.J.S.), who excluded cases without objective imaging finding of PE or PEs that were (1) not acute, (2) not diagnosed during the ED evaluation, or (3) not deemed to be clinically significant or treated. Medical record review followed published guidelines,^19^ including use of a standardized data abstraction form (Supplement 2), which was pilot tested and refined; blinding of reviewers to study hypothesis; and assessment of interrater reliability for key data elements. Disagreements were adjudicated by another of us (C.F.G.). Mortality at 7 and 30 days after PE diagnosis was verified using the state of Michigan Death Index. We followed the Strengthening the Reporting of Observational Studies in Epidemiology (STROBE) guidelines.^20^

Race and ethnicity were self-reported with the following categories: American Indian and Alaska Native, Asian, Black or African American, Native Hawaiian or other Pacific Islander, White, unknown, other, and patient refused. Race and ethnicity were included in our analysis to help describe the diversity of the population studied so that future studies can compare and assess how population differences may impact their results

### PESI Score or Class and Risk Stratification

Abstractors recorded any PESI score or class documented in the patient medical record and also calculated the PESI score using abstracted variables and peak vital signs during the ED stay. Interrater reliability was determined for the calculated PESI class, and in the event of disagreement, an adjudicating review was performed.

### Blood-Based Biomarkers

Troponin I and high-sensitivity troponin T (our hospital system transitioned to the latter assay in March 2018) were included in the registry when measured as part of the ED evaluation. For the subset of acute ED-PEs for which biomarkers were available, we performed a parallel analysis in which patients with abnormal troponin levels were classified as high-risk irrespective of PESI class.

### CTPE Findings

CTPE findings were abstracted from the radiology reading, including laterality (bilateral vs unilateral), largest vessel involved (saddle, main, lobar, segmental, subsegmental), RV-to–left ventricle (LV) ratio, and presence of RV enlargement, septal abnormality (eg, flattening, straightening, bowing), or pulmonary infarct. Missing data were obtained by fresh review of CTPE images by 2 board-certified radiologists (A.L.L. and M.S.K.). Patients were classified as having concerning CT imaging findings if 1 or more were present: (1) bilateral embolus described as saddle or main pulmonary arteries, (2) RV-LV ratio greater than 1, (3) RV enlargement, (4) septal abnormality consistent with RV pressure overload (eg, flattening, straightening, bowing), or (5) pulmonary infarction.

### Statistical Analysis

Bivariate linear and logistic regression analyses were used to test hypotheses about risk-group differences in continuous and categorical outcomes, respectively. For continuous outcomes, effect sizes were calculated as unstandardized between-group differences. For categorical outcomes, effect sizes were calculated as unstandardized marginal effects (eg, differences in percentages). An α level of .05 was used for all analyses, and all hypothesis tests were 2-sided. Analyses were conducted with the Stata statistical software package version 15 (StataCorp). Statistical analysis was performed in June to October 2022.

## Results

### Construction of the PE Registry

Construction of the acute ED-PE registry is shown in flow sheet form in eFigure 1 in Supplement 1. Briefly, of 10 671 patient encounters in which a CTPE was performed during or just prior to ED evaluation, 967 (90.6%) were identified as positive PE cases by MEDIC abstractors or EMR query. Physician review of these 967 medical records led to exclusion of 63 cases (6.5%) that were deemed not to have objective evidence of PE and an additional 87 cases (8.9%) in which the PE was either chronic, septic, deemed clinically insignificant, diagnosed after the patient had left the ED, or previously diagnosed and treated at another facility. Interrater reliability for the diagnosis of acute ED-PE was high (Cohen κ = 0.79). There were 21 patients with multiple entries; in each case, physician review confirmed that each entry represented a distinct and acute thromboembolic event based on CTPE findings and initiation of new treatment. Ultimately, 817 acute ED-PEs were included in the registry: 417 (51.0%) in women and 400 (49.0%) in men. The mean (IQR) patient age was 58 (47-71) years. Demographic and clinical variables were similar for patients with acute-ED PEs vs non-PEs vs nonacute/nonsignificant PEs (eTable in Supplement 1), with the except of ED disposition. Only 21 of 817 patients with acute ED-PE (2.6%) were treated as outpatients (not including those patients sent home on hospice), while 22% of patients in the non-PE (14 of 63) and nonacute or nonsignificant PE (19 of 87) groups were discharged.

### PESI Score and Risk Stratification

**Figure 1.**
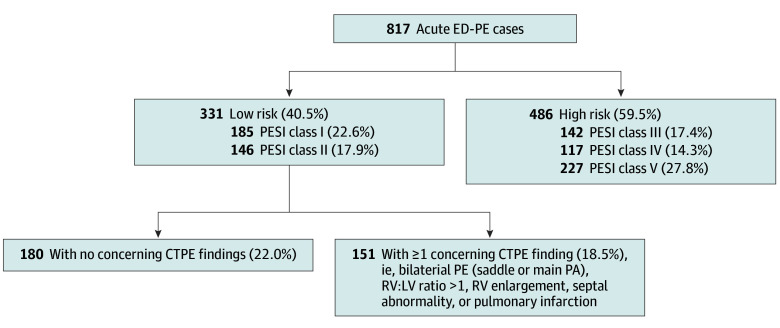
Risk Stratification of Acute Pulmonary Embolisms Diagnosed in the Emergency Department (ED-PEs) Acute ED-PE cases were divided into low- and high-risk groups based on Pulmonary Embolism Severity Index (PESI) score or class. As in previous reports, cases were spread fairly evenly among the 5 risk classes. A higher proportion was seen in the highest risk class, reflecting the high acuity and rate of comorbidities in the patient population in this study. The low-risk group was further divided based on the presence or absence of 5 concerning findings on pulmonary embolism–protocol computed tomography (CTPE; ie, findings widely believed to confer higher risk). LV indicates left ventricle; PA, pulmonary artery; RV, right ventricle.

Figure 1 shows the distribution of the 817 acute ED-PEs among the 5 PESI classes: 331 (40.5%) were in class I or class II (hereafter referred to as low-risk), while 486 (59.5%) were in classes III to V (hereafter referred to as high-risk). Interrater reliability for the calculated PESI class was very high (Cohen κ = 0.83). Moreover, the calculated value matched the documented PESI score or class in more than 90% of cases (52 of 56) in which PESI score or class was documented by ED clinicians. The Table compares demographic variables and components of the PESI score for low- and high-risk groups.

**Table.** Demographic Characteristics, PESI Score Components, and CTPE Findings in Patients Low- vs High-risk Acute ED-PEs and in Low-risk Patients With and Without Concerning CTPE Findings

Characteristic	Acute ED-PEs			Low-risk PEs		
	Low-risk PEs (n = 331)	High-risk PEs (n = 486)	*P* value	No concerning CTPE findings (n = 180)	≥1 Concerning CTPE findings (n = 151)	*P* value
Demographic characteristics						
Age, median (IQR), y	46 (34-57)	66 (57-76)	<.001	45 (33-57)	47 (35-59)	.31
Sex						
Male, No. (%)	152 (45.9)	248 (51.0)	.16	77 (42.8)	75 (49.7)	.22
Female	179 (54.1)	238 (49.0)		103 (57.2)	76 (50.3)	
Race, No. (%)						
Black or African-American	70 (21.1)	59 (12.1)	.001	42 (23.3)	28 (18.5)	.31
White	240 (72.5)	405 (83.3)	<.001	132 (73.3)	108 (71.5)	.72
Other or refused^a^	21 (6.3)	22 (4.5)	.27	6 (3.3)	15 (9.9)	.02
Ethnicity, No. (%)						
Hispanic	7 (2.1)	8 (1.6)	.63	4 (2.2)	3 (2.0)	.88
Non-Hispanic or refused	324 (97.9)	478 (98.4)	.63	176 (97.8)	148 (98.0)	.88
PESI score						
Health history, No. (%)						
Active cancer	17 (5.1)	248 (51.0)	<.001	15 (8.3)	2 (1.3)	.002
Heart failure	10 (3.0)	61 (12.6)	<.001	9 (5.0)	1 (0.7)	.02
Chronic lung disease	41 (12.4)	117 (24.1)	<.001	28 (15.6)	13 (8.6)	.06
Peak vitals, mean (IQR)						
Highest HR, beats/min	95 (85-105)	110 (93-126)	<.001	95 (85-106)	94 (84-102)	.66
Highest RR, breaths/min	20 (18-22)	28 (20-31)	<.001	20 (18-20)	20 (18-22)	.42
Lowest SBP, mm Hg	123 (111-135)	111 (95-125)	<.001	122 (109-134)	124 (112-135)	.30
Lowest temperature, °C	36.8 (36.6-36.9)	36.5 (36.4-36.8)	<.001	36.8 (36.5-36.9)	36.8 (36.6-36.9)	.47
Lowest SpO_2_, %	95 (94-97)	89 (88-95)	<.001	96 (94-97)	95 (93-97)	.004
Supplement O_2 _>2L, No. (%)^b^	14 (4.2)	182 (37.4)	<.001	7 (3.9)	6 (4.0)	.74
PESI score, mean (IQR)	60 (46-74)	133 (102-155)	<.001	60 (47-75)	59 (46-71)	.33
CTPE findings						
Laterality, No. (%)						
Bilateral	181 (54.7)	250 (51.4)	.36	67 (37.2)	114 (75.5)	<.001
Unilateral	150 (45.3)	236 (48.6)	.36	113 (62.8)	37 (24.5)	<.001
Largest artery involved, No. (%)						
Saddle	7 (2.1)	20 (4.1)	.12	0	7 (4.6)	.004
Main	51 (15.4)	100 (20.6)	.06	2 (1.1)	49 (32.5)	<.001
Lobar	74 (22.4)	107 (22.0)	.91	35 (19.4)	39 (25.8)	.16
Segmental	145 (43.8)	191 (39.3)	.20	99 (55)	46 (30.5)	<.001
Subsegmental	54 (16.3)	68 (14.0)	.37	44 (24.4)	10 (6.6)	<.001
RV-LV ratio, mean (IQR)	0.99 (0.80-1.10)	1.11 (0.88-1.30)	<.001	0.83 (0.79-0.9)	1.11 (0.92-1.20)	<.001
Enlarged RV, No. (%)	35 (10.6)	73 (15.0)	.06	0	35 (23.2)	<.001
Septal abnormality, No. (%)^c^	83 (25.1)	147 (30.2)	.10	0	83 (55)	<.001
Pulmonary infarct, No. (%)	62 (18.7)	63 (13.0)	.03	0	61 (40.4)	<.001

Abbreviations: CTPE, pulmonary embolism–protocol computed tomography; ED-PE, pulmonary embolism diagnosed in the emergency department; HR, heart rate; LV, left ventricle; PE, pulmonary embolism; PESI, Pulmonary Embolism Severity Index; RR, respiratory rate; RV, right ventricle; SBP, systolic blood pressure; SpO_2_, oxygen saturation.

^a^ Other included American Indian and Alaska Native, Asian, Native Hawaiian and Pacific Islander, unknown, and other.

^b^ Supplemental oxygen of more than 2 L per minute or more than 2 L per minute greater than the patient’s baseline for patients receiving home O_2_.

^c^ Septal abnormalities includes flattening, straightening, or bowing indicative of right heart strain.

### CTPE Findings in Patients With Acute Low- and High-risk ED-PE

We next examined the frequency of various CTPE findings in low- and high-risk groups (Table). There were no statistically significant differences in the frequency of bilateral PEs (low-risk: 181 [54.7%]; high-risk: 250 [51.4%]; difference, −1.7%; 95% CI, −8.8% to 5.4%; *P* = .36). However, more high-risk cases were saddle PEs or involved the main pulmonary arteries vs low-risk cases (120 [24.7%] vs 58 [17.5%]; difference, 7.2%; 95% CI, 3.5% to 10.8%; *P* = .01). Likewise, high-risk cases had statistically higher mean (IQR) RV-LV ratios (1.11 [0.88 to 1.30] vs 0.99 [0.80-1.10]; mean difference, 0.12; 95% CI 0.08 to 0.02; *P* < .001). There was no statistically significant difference between groups for RV enlargement or septal abnormalities, but more patients in the high-risk group vs the low-risk group had these findings (RV enlargement: 73 [15.0%] vs 35 [10.6%]; difference, 4.4%; 95% CI, 2.2% to 6.5%; *P* = .06; septal abnormalities: 147 [30.2%] vs 83 [25.1%]; difference, 5.1%; 95% CI, 2.1% to 8.1%; *P* = .10). Pulmonary infarction was less common in the high-risk group vs the low-risk group (63 [13.0%] vs 62 [18.7%]; difference, −5.7%; 95% CI, −0.6% to −11.1%; *P* = .03).

### Short-term Clinical Outcomes in Patients With Acute Low-risk ED-PE With and Without Concerning CTPE Findings

**Figure 2.**
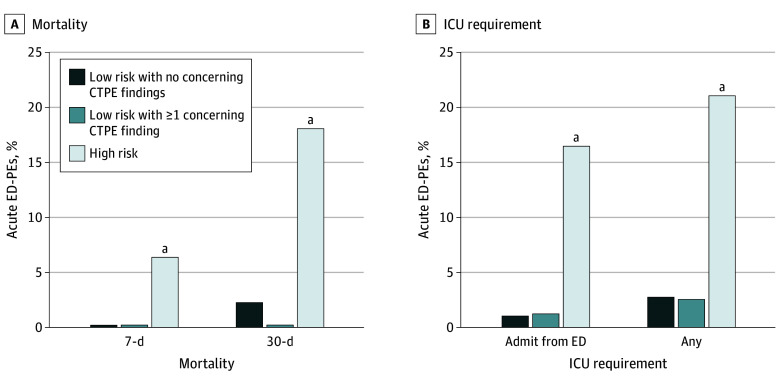
Clinical Outcomes and Findings on Pulmonary Embolism–Protocol Computed Tomography (CTPE) A. All-cause mortality at 7 and 30 days was similar for patients with low-risk pulmonary embolism diagnosed in the emergency department (ED-PE) irrespective of CTPE findings, but significantly higher for high-risk ED-PE. B. Few patients with low-risk pulmonary embolism required admission into the intensive care unit (ICU), while patients with high-risk pulmonary embolism had higher frequency of ICU admission, both directly from the ED and at any time during hospitalization. Box and whiskers show 25th to 75th percentiles and 10th to 90th percentiles, respectively. ^a^*P* < .001.

As shown in Figure 1, we further divided the low-risk group based on the absence (180 [54.3%]) or presence (151 [46.7%]) of 1 or more concerning CTPE findings. The Table shows comparisons of demographic, clinical, and radiographic variables for these groups, while Figure 2 shows short-term clinical outcomes. There were no deaths at 7 days in either low-risk group, whereas 7-day mortality was 6.8% (33 of 486) in the high-risk group (difference, −6.8%; 95% CI, −3.7% to −9.4%). At 30 days, mortality remained quite low in both low-risk groups vs 18.1% morality (88 patients) in the high-risk group (high-risk vs low-risk group with concerning CTPE findings: difference, 18.1%; 95% CI, 14.1% to 21.8%; *P* < .001). Even low-risk acute ED-PEs with the most concerning appearing CTPEs, eg, 41 cases with 3 concerning findings of bilateral, central emboli with an RV-LV ratio greater than 1 and septal abnormalities, had no deaths at 30 days.

Very few patients treated in the ED from either low-risk group required admission to the intensive care unit (ICU) (without concerning findings: 2 [1.1%]; with concerning findings: 2 [1.3%]; difference, 0.2%; 95% CI, −2.6% to 2.2%; *P* = .86). Both low-risk groups were significantly different from the high-risk group, in which 80 patients (16.5%) were admitted from ED directly to ICU (difference, 15.2%; 95% CI, 11.4% to 18.9%; *P* < .001). The pattern remained unchanged when considering requirement for ICU-level care at any time during hospitalization: 4 (2.6%) and 5 (2.8%) for low-risk groups with and without concerning CTPE findings, respectively (difference, 0.2%; 95% CI, −3.3% to 3.6%; *P* = .94) and 103 (21.1%) for the high-risk group (difference, 18.5%; 95% CI, 14.0% to 23.0%; *P* < .001). Even multiple concerning CTPE findings for an individual patient did not have a significant association with ICU utilization in low-risk patients, with only 2 of 55 patients (3.6%) with 2 or more concerning CTPE findings requiring ICU-level care.

### CTPE Findings and Hospitalization of Patients With Low-risk Acute ED-PE

**Figure 3.**
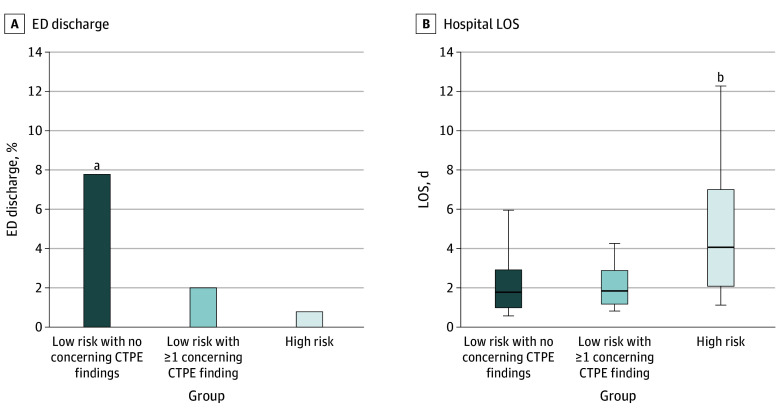
Hospitalization and Findings on Pulmonary Embolism–Protocol Computed Tomography (CTPE) A, The frequency of outpatient management, represented by emergency department (ED) discharge, was higher for patients with low-risk pulmonary embolisms without concerning CTPE findings. B, Hospital length of stay (LOS) for patients who were admitted, which was similar among low-risk patients, but significantly longer for high-risk patients. Box and whiskers show 25th to 75th percentiles and 10th to 90th percentiles, respectively. ^a^*P* = .01. ^b^*P* < .001.

Figure 3 shows the rate of ED discharge and the mean hospital length-of-stay (LOS) for each group of patients with acute ED-PEs. There was a higher rate of outpatient treatment in low-risk patients in the absence of concerning CTPE findings vs those with concerning CTPE findings (14 [7.8%] vs 3 [2.0%]; difference, 5.8%; 95% CI, 1.3% to 10.3%; *P* = .01), with no significant difference between the low-risk group with concerning CTPE findings and the high-risk group (2.0% vs 4 [0.8%]; difference, 1.2%; 95% CI, −1.2% to 3.5%; *P* = .34). The pattern was reversed for hospital LOS, which was a mean (SD) of 2.3 (1.9) and 2.6 (3.5) days for the low-risk groups with and without concerning CTPE findings, respectively (difference, 0.3 days; 95% CI, −0.3 to 0.9 days; *P* = .32), and 5.8 (5.9) days for the high-risk group (vs low-risk group with concerning CTPE findings: difference, −3.5 days; 95% CI, −4.2 to −2.9; *P* < .001).

### CTPE Findings and Resource Utilization in Low-risk Acute ED-PE

**Figure 4.**
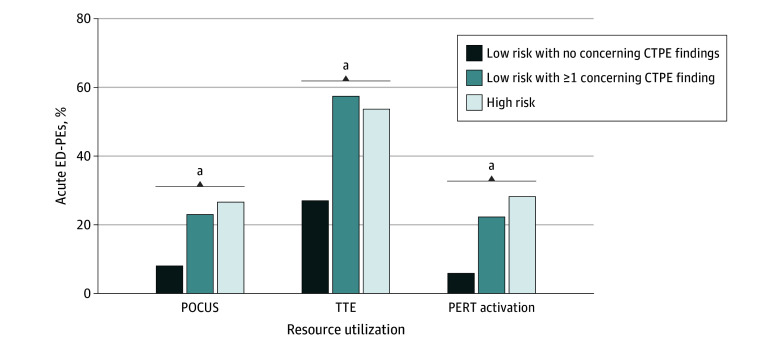
Resource Utilization and Findings on Pulmonary Embolism–Protocol Computed Tomography (CTPE) Frequency of point-of-care ultrasonography (POCUS) and transthoracic echocardiography (TTE) was significantly lower for low-risk pulmonary embolisms without concerning CTPE findings vs low-risk pulmonary embolisms with concerning findings and high-risk pulmonary embolisms. Similarly, activation of the Pulmonary Embolism Response Team (PERT), a multidisciplinary consult service was less frequent in low-risk pulmonary embolisms without concerning findings. ^a^*P* < .001.

Both cardiac point-of-care ultrasonography (POCUS) and formal transthoracic echocardiography (TTE) were performed more frequently in low-risk cases with than without concerning CTPE findings: 35 (23.2%) vs 15 (8.3%) for POCUS (difference, 14.9%; 95% CI, 6.9%-22.8%; *P* < .001) and 87 (57.6%) vs 49 (27.2%) for TTE (difference, 30.4%; 95% CI, 20.1%-40.7%; *P* < .001). Similarly, the multidisciplinary PE response team (PERT), which advises ED clinicians in cases in which they believe advanced therapies (eg, catheter-directed thrombolysis) might be beneficial, was activated in 34 (22.5%) low-risk cases with concerning CTPE findings vs 11 (6.1%) without (difference, 16.4%; 95% CI, 8.8%-24.0%, *P* < .001). The frequency of POCUS (130 [26.8%]), TTE (262 [53.9%]), and activation of the PERT (138 [28.4%]) in the high-risk group were not statistically different compared with the low-risk group with concerning CTPE findings (Figure 4).

### Biomarkers

Overall, 709 cases (86.8%) had at least 1 troponin level measured as part of the ED evaluation. We performed a parallel analysis (eFigure 2 in Supplement 1), defining low-risk as PESI class I to II with reference-range biomarker levels and high-risk as PESI class III to V with abnormal troponin levels. This produced similar findings, with no major differences in clinical outcomes or patterns of hospitalization or resource utilization across the risk groups (eFigure 3 in Supplement 1).

## Discussion

The results of the current study indicate that CTPE findings widely believed to confer high risk in the setting of acute PE are associated with increased hospitalization and resource utilization in ED patients with low risk, but they were not associated with adverse clinical outcomes. These findings were remarkably consistent across our relatively large registry of acute ED-PE cases and persisted whether low risk was defined based on PESI class alone or a combination of PESI and biomarker results. Impressively, even multiple concerning CTPE findings and combinations that EM physicians have been trained to fear (eg, saddle PE with right heart strain) had no obvious association with short-term mortality or need for intensive care in otherwise low-risk patients.

At first glance, our results seem to conflict with recent reports in the field. Specifically, there have been 2 meta-analyses^17,18^ published in the past several years that concluded that RVD is associated with short-term mortality in low-risk PE. Both reports, however, pooled data from studies using different measures of RVD, and their primary conclusions were based on combinations of CTPE, echocardiography, and biomarker testing. In the one case in which imaging modalities and biomarkers could be separated—due to an individual patient-data meta-analysis (IPDMA) method—RVD assessed via CTPE was not found to be associated with in-hospital or 30-day mortality. Of note, this pooled IPDMA population also matched our cohort fairly well in terms of the age, comorbidities, rate of concerning CTPE findings, and overall 30-day mortality among low-risk patients.^18^

While our findings require confirmation, it is worth considering their potential impact on EM practice. Over the past 2 decades, a strong evidence base has been established to support safe outpatient management of PE, but adoption by EM clinicians has been slow and remains limited in most settings.^3,7,8,21,22,23^ A number of implementation studies have been conducted, focusing on creation of tools for electronic clinical decision support and protocols to facilitate identification, appropriate management, and timely follow-up of low-risk patients.^4,5,24^ These studies, however, have either explicitly excluded patients with concerning CTPE findings or simply not addressed the potential association between perceived risk and physician decision-making. Our findings suggest not only that outpatient management may be considered for these patients, but that concerning CTPE findings represent an important barrier to discharge. Compelling evidence, including the results of the current study, will likely need to be paired with effective implementation strategies to overcome their perceived risk. Indeed, as clinicians and health systems become more comfortable and facile with other aspects of home treatment, we expect a widening of the gap in discharge of low-risk patients with and without concerning CTPE findings.

Beyond clinical implications, our study has interesting aspects from the stand point of PE research and quality improvement. In particular, construction of the acute ED-PE registry required a multimodal approach, combining manual medical record abstraction (by the MEDIC collaborative), query of the EMR, and a substantial amount of physician medical record review to identify and exclude inappropriate cases. Nonacute and nonsignificant PEs were often difficult to distinguish from acute ED-PEs without physician review of the documented medical decision-making. For example, patients who presented to our facility for second opinion after recent diagnosis and treatment at another hospital were treated quite differently by EM clinicians, as evidenced by the greater than 10-fold higher rate of discharge, but typically had acute-appearing emboli on imaging and final clinical impressions of acute PE. Similarly, identification and separation of insignificant subsegmental PEs, radiographic mimics (eg, infiltrating tumors), and chronic PEs are likely to remain a major challenge for automated EMR queries or non–medically trained abstractors. Our approach with repeated physician reviews resulted in a high-fidelity registry with essentially no missing data and high confidence in the accuracy and acuity of each PE diagnosis. At the same time, the experience raises concerns about the accuracy of PE data acquired from national databases or unfiltered EMR queries. These observations may be particularly important as health systems and researchers create interventions to evaluate and increase outpatient PE treatment.

### Limitations

Our study does have some limitations. The primary outcome was uncommon, so our study may be underpowered to detect modest differences between groups. The study was also retrospective and limited to a single center. These limitations are mitigated, to some extent, by the relatively large number of cases in the registry and the numerous steps taken to minimize issues typically associated with retrospective medical review, eg, repeated physician review of each medical record, radiology overread of CTPE scans with missing measurements, and so on. These measures aside, retrospective design limits the ability to discern what factors influenced physician behavior at the time of patient care and introduces the possibility of confounding variables that may not have been accounted for, eg, social and economic factors requiring admission. Similarly, our practice setting—an academic, tertiary health care center with a large population of patients with advanced cancer and cardiopulmonary disease—does not reflect all hospitals, and our cohort had limited racial and ethnic diversity. To be truly generalizable, our findings will need to be reproduced in prospective, multicenter studies encompassing the full spectrum of EM practice. Our study is also somewhat limited in its focus on concerning CTPE findings as opposed to ultrasonographic findings. While many patients in our registry underwent formal TTE, it was infrequently completed during ED evaluation, and POCUS was limited by inconsistency in the training of operators and quality of documentation. Our analysis was also limited to short-term clinical outcomes, and it is possible that CTPE findings could be associated with other clinically significant endpoints, eg, post-PE syndrome, recurrent venous thromboembolism, or chronic thromboembolic pulmonary hypertension. Furthermore, our study does not address risk of bleeding, although other studies have consistently shown low rates of major bleeding in low-risk patients with acute ED-PE.

## Conclusions

In summary, ED patients with acute, low-risk PE had similar short-term outcomes irrespective of CTPE results. Nonetheless, specific CTPE findings were associated with increased resource utilization and hospitalization of these patients. Future implementation studies aimed at maximizing guideline-recommended outpatient management of acute low-risk PE should account for and address EM clinician concerns regarding CTPE imaging.

## Notes

Supplement 1. **eMethods. **Supplemental Methods **eFigure 1.** Construction of Acute PE Registry **eTable.** Acute ED-PE Patient Characteristics and ED Disposition **eFigure 2.** Risk Stratification of Acute ED-PEs Incorporating PESI Score and Troponins **eFigure 3.** Association of Clinical Outcomes, Hospitalization, and Resource Utilization With CTPE Findings, Incorporating PESI Score and Troponins

Supplement 2.
Data Abstraction Form

Supplement 3.
Data Sharing Statement
