# Error in Table

**DOI:** 10.1001/jamanetworkopen.2024.43500

**Published:** 2024-10-10

In the Original Investigation titled “Adverse Clinical Outcomes Among Patients With Acute Low-Risk Pulmonary Embolism and Concerning Computed Tomography Imaging Findings,”^1^ published May 31, 2023, there was an error in the Table. The number of patients with high-risk pulmonary embolisms diagnosed in the emergency department whose largest involved artery was subsegmental should have read 68. This article has been corrected.^1^

## References

[1] O’Hare C, Grace KA, Schaeffer WJ, . Adverse clinical outcomes among patients with acute low-risk pulmonary embolism and concerning computed tomography imaging findings. JAMA Netw Open. 2023;6(5):e2311455. doi:10.1001/jamanetworkopen.2023.11455 37256624 PMC10233419

