# Supplementary material for: Adverse Clinical Outcomes Among Patients With Acute Low-risk Pulmonary Embolism and Concerning Computed Tomography Imaging Findings
Source: JAMA Netw Open. 2023 May 31;6(5):e2311455. doi: 10.1001/jamanetworkopen.2023.11455 (PMC10233419; doi:10.1001/jamanetworkopen.2023.11455)
Supplement: Supplement 1. — eMethods. Supplemental Methods eFigure 1. Construction of Acute PE Registry eTable. Acute ED-PE Patient Characteristics and ED Disposition eFigure 2. Risk Stratification of Acute ED-PEs Incorporating PESI Score and Troponins eFigure 3. Association of Clinical Outcomes, Hospitalization, and Resource Utilization With CTPE Findings, Incorporating PESI Score and Troponins [file jamanetwopen-e2311455-s001.pdf]

Supplemental Online Content

O'Hare C, Grace KA, Schaeffer WJ, et al. Adverse clinical outcomes among patients with acute low-risk pulmonary embolism and concerning computed tomography imaging findings. *JAMA Netw Open.* 2023;6(5):e2311455.
doi:10.1001/jamanetworkopen.2023.11455

eMethods. Supplemental Methods

eFigure 1. Construction of Acute PE Registry

eTable. Acute ED-PE Patient Characteristics and ED Disposition

eFigure 2. Risk Stratification of Acute ED-PEs Incorporating PESI Score and Troponins

eFigure 3. Association of Clinical Outcomes, Hospitalization, and Resource Utilization With CTPE Findings, Incorporating PESI Score and Troponins

This supplemental material has been provided by the authors to give readers additional information about their work.

© 2023 O'Hare C et al. *JAMA Network Open.*

eMethods. Supplemental Methods

Construction of the U-M acute ED-PE registry

Construction of the U-M registry began with data from the Michigan Emergency Department Improvement Collaborative (MEDIC), a state-wide quality Collaborative Quality Initiative (CQI) which reviews the charts of all patients who undergo CTPE in one of the 37 Emergency Departments within the network. Positive PE cases from our institution, as identified by MEDIC abstractors, were pooled with those identified by query of our electronic medical record. The latter was done, in part, to capture patients diagnosed on outpatient CTPE who were immediately referred to the ED. Of the 967 cases identified by MEDIC abstractors and EMR query (Supplemental Figure 1), physician reviewers excluded 63 cases in which there was no objective evidence of PE. The most common reason for exclusion was inaccuracy in MEDIC abstraction (24 cases). Reviewers also excluded 14 cases that had been inappropriately identified as PEs via query of the EMR, 3 cases in which a diagnosis of PE was made presumptively by the ED provider (based on high clinical suspicion) but CTPE imaging was ultimately negative, and 22 cases in which the initial radiology read indicated PE, but final interpretation was negative (motion artifact, 5 cases; inadequate contrast bolus, 5 cases; infiltrating tumor, 5 cases; low flow due to severe pulmonary hypertension, 2 cases; misinterpretation by a radiology trainee, 5 cases). An additional 87 cases (8.9%) were excluded due to the PE being chronic (58 cases), not clinically significant (all isolated subsegmental PEs which were not treated, 7 cases), septic emboli (2 cases), diagnosed after the patient had left the ED (5 cases), or previously diagnosed and treated at another facility (15 cases).

Chart abstraction and data verification

For each chart identified as an acute ED-PE, demographic data (age, sex, ethnicity, date of birth, date/time of ED arrival, date/time of ED or hospital discharge), vital signs, and laboratory results were electronically abstracted from the electronic medical record. Missing values were obtained via manual chart review. Additional data elements were manually abstracted by two physician reviewers, including components of the PESI score (see below), CTPE findings (see below), ultrasonography (point-of-care ultrasonography and formal transthoracic echocardiography), treatment (anticoagulation, PE Response Team [PERT] activation, use of thrombolytics and/or advanced interventions, and hospitalization), and outcomes (need for intensive care and mortality). PERT activation was analyzed only for acute ED-PEs which occurred after June 1st, 2017, when the PERT team was formed. Disagreements were adjudicated by third physician review.

Calculation of PESI score/class from abstracted variables

PESI score and class were calculated using abstracted variables - age, gender, comorbidities, mental status, and peak vital signs during the ED stay (i.e., maximum heart rate, maximum respiratory rate, minimum systolic blood pressure, and lowest O_2 saturation). In assessing history of cancer (+30 points), only active cancers were counted. Patients were considered hypoxic (+20 points) if they did not have a history of chronic (i.e., home) oxygen use and required supplemental O_2 of > 2L, or if they required > 2L increase over their home oxygen requirement.

© 2023 O'Hare C et al. *JAMA Network Open.*

Blood-based Biomarkers

While precise cut-offs for Troponin I (TnI) and high-sensitivity Troponin T (hs-TnT) have not been defined in the context of acute ED-PE, we reasoned that EM providers would be unlikely to consider any patient with an abnormal troponins (as defined by our hospital laboratory) to be low risk. With this in mind, values above the 99th percentile for our laboratory (> 0.16 ng/mL for TnI, > 19 ng/L for hs-TnT, and > 100 pg/mL for BNP) were considered abnormal.

© 2023 O'Hare C et al. *JAMA Network Open.*

eFigure 1. Construction of Acute PE Registry

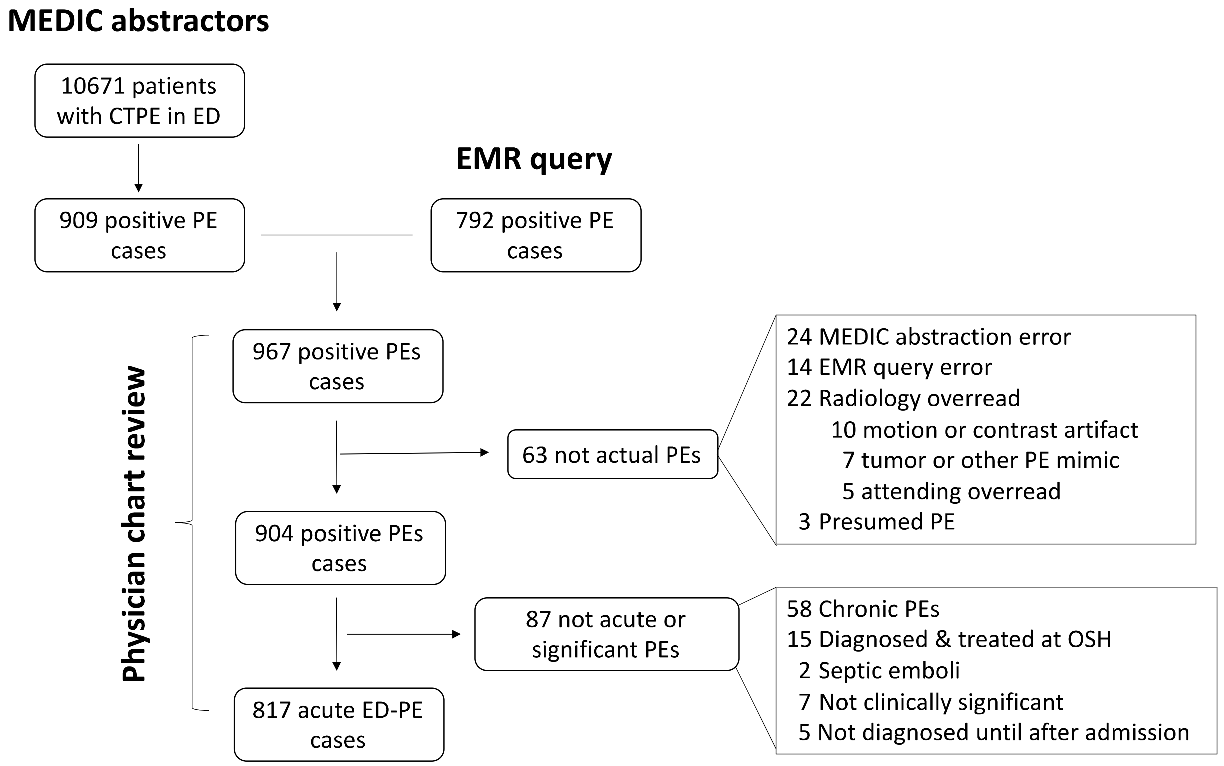

© 2023 O'Hare C et al. *JAMA Network Open.*

eTable. Acute ED-PE Patient Characteristics and ED Disposition

	Acute ED-PE (n=817)	Non-PE (n=63)	Non-acute or significant PE (n=87)
**Sex, n (%)**			
Female	417 (51)	34 (52)	47 (55)
Male	400 (49)	31 (48)	38 (45)
**Age, years [IQR]**	58 [47, 71]	58 [46, 73]	60 [48, 71]
**Race, n (%)**			
White or caucasian	645 (79)	50 (78)	66 (78)
Black or African-American	129 (16)	12 (18)	12 (14)
Asian	17 (2)	0 (0)	3 (3)
Native American or Alaskan	2 (<1)	1 (1)	0 (0)
Hawaiian/Pacific Islander	1 (<1)	0 (0)	0 (0)
Other/Refused	23 (3)	2 (3)	4 (4)
**Ethnicity, n (%)**			
Hispanic	15 (2)	0 (0)	2 (2)
Non-Hispanic	792 (97)	65 (100)	82 (97)
Refused	10 (1)	0 (0)	1 (1)
**ED Disposition**			
Admission	785 (96)	47 (72)	64 (75)
Discharge	21 (3)	14 (22)	19 (22)
Deceased/hospice	9 (1)	1 (1)	0 (0)
Other*	1 (<1)	3 (5)	2 (3)

Acute ED-PEs had similar demographics as compared to excluded cases. However, the rate of outpatient management for acute ED-PEs was markedly different than for non-PEs (2.6% vs. 22.2%, p<0.001) or non-acute or non-significant PEs (2.6% vs. 21.8%, p<0.001). *Other indicates patients who left AMA or were sent to OB triage or Psychiatric Emergency Services.

© 2023 O'Hare C et al. *JAMA Network Open.*

eFigure 2. Risk Stratification of Acute ED-PEs Incorporating PESI Score and Troponins

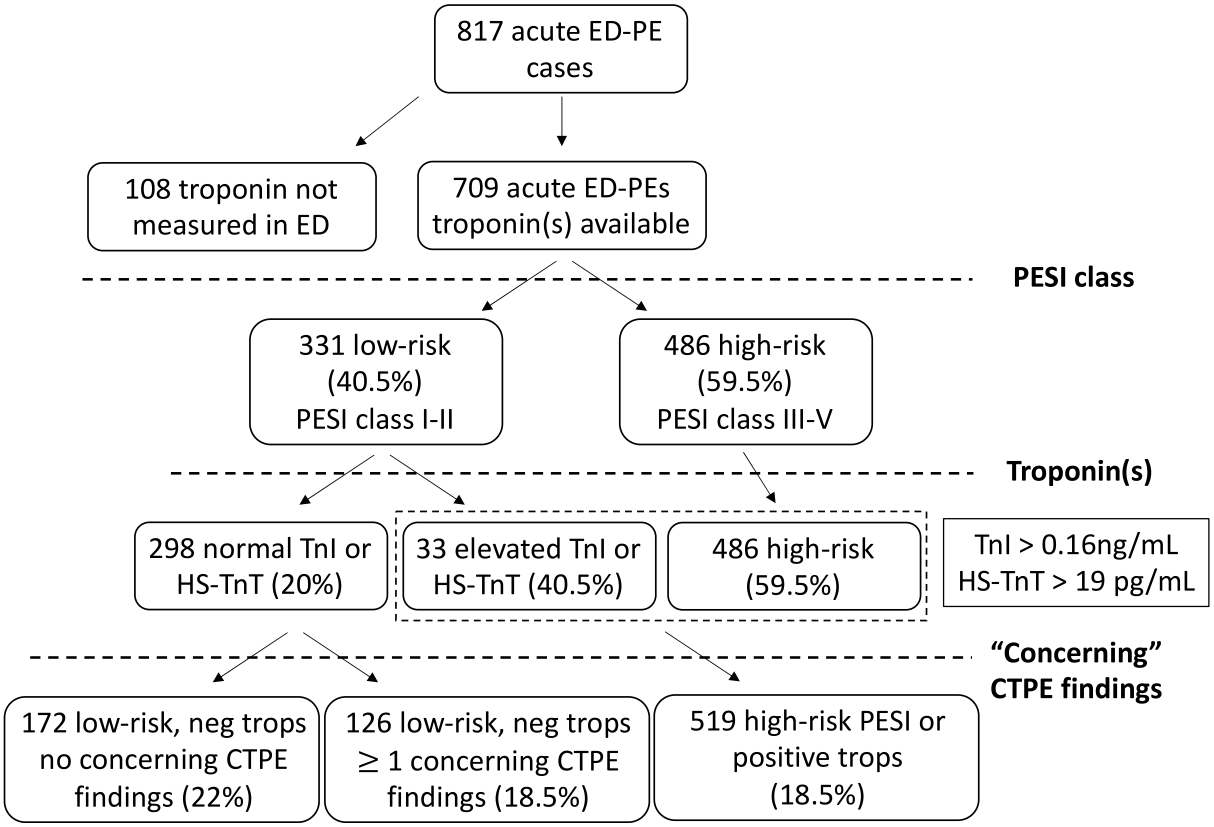

Alternate analysis for the 709 acute ED-PE cases in which troponins were measured as part of ED evaulation. Following risk-stratification by PESI score/class, 33 low-risk cases were reclassified based on elevated TnI or hs-TnT. The remaining low-risk cases were then divided based on presence or absence of "concerning" CTPE findings.

© 2023 O'Hare C et al. *JAMA Network Open.*

eFigure 3. Association of Clinical Outcomes, Hospitalization, and Resource Utilization With CTPE Findings, Incorporating PESI Score and Troponins

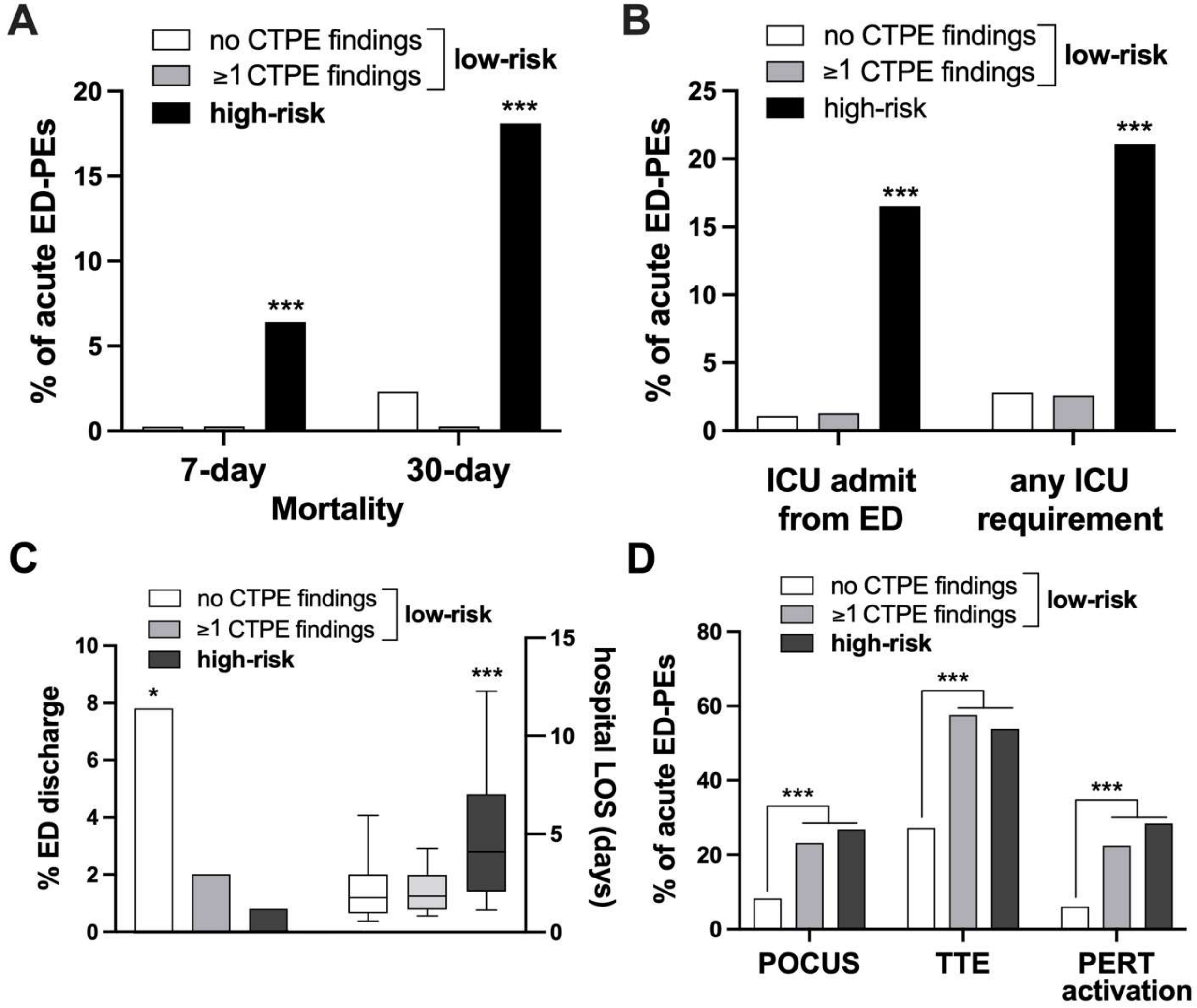

Results of parallel analysis incorporating PESI score/class and biomarkers into risk stratification. The associations of CTPE findings with clinical outcomes, hospitalization, and resource utilization in low-risk acute ED-PEs remained unchanged. A. 7- and 30-day mortality, *** - p < 0.001 vs. both low-risk groups, B. need for intensive care, *** - p < 0.001 vs. both low-risk groups, C. Rate of outpatient management (* - p = 0.01) and hospital length of stay (LOS, ** - p < 0.01), D. point of care ultrasound (POCUS), transthoracic echocardiography (TTE), and PE response team activation (PERT), *** - p < 0.001.

© 2023 O'Hare C et al. *JAMA Network Open.*
