# Supplementary material for: Adverse Clinical Outcomes Among Patients With Acute Low-risk Pulmonary Embolism and Concerning Computed Tomography Imaging Findings
Source: JAMA Netw Open. 2023 May 31;6(5):e2311455. doi: 10.1001/jamanetworkopen.2023.11455 (PMC10233419; doi:10.1001/jamanetworkopen.2023.11455)
Supplement: Supplement 3. — Data Sharing Statement [file jamanetwopen-e2311455-s003.pdf]

Data Sharing Statement

O'Hare. Adverse Clinical Outcomes Among Patients With Acute Low-risk Pulmonary Embolism and Concerning Computed Tomography Imaging Findings. *JAMA Netw Open*. Published May 31, 2023. doi:10.1001/jamanetworkopen.2023.11455

Data
Data available: Yes
Data types: Deidentified participant data, Data dictionary
How to access data: coling@med.umich.edu
When available: With publication

Supporting Documents
Document types: None

Additional Information
Who can access the data: researchers whose proposed use of the data has been approved
Types of analyses: for a specified purpose
Mechanisms of data availability: with a signed data access agreement
